# A Silicone Oil-Free Syringe Tailored for Intravitreal Injection of Biologics

**DOI:** 10.3389/fopht.2022.882013

**Published:** 2022-05-04

**Authors:** Torleif T. Gjølberg, Heidrun E. Lode, Gustavo B. Melo, Simone Mester, Christine Probst, Magne S. Sivertsen, Øystein K. Jørstad, Jan Terje Andersen, Morten C. Moe

**Affiliations:** ^1^ Department of Immunology, Oslo University Hospital Rikshospitalet, Oslo, Norway; ^2^ Department of Pharmacology, Oslo University Hospital and University of Oslo, Oslo, Norway; ^3^ Department of Ophthalmology, Oslo University Hospital and University of Oslo, Oslo, Norway; ^4^ Department of Ophthalmology, Federal University of São Paulo, São Paulo, Brazil; ^5^ Department of Ophthalmology, Hospital de Olhos de Sergipe, Aracaju, Brazil; ^6^ Department of Research and Development Sciences, Luminex Corporation, Seattle, WA, United States

**Keywords:** intravitreal injection, anti-VEGF, silicone oil, compounding, AMD, syringe

## Abstract

Intravitreal injections (IVI) of biologics targeting vascular endothelial growth factor (anti-VEGF) led to a paradigm shift in the management and prognosis of prevalent retinal conditions. Yet, IVI are typically performed with syringes that are neither developed nor approved for this purpose. Notably, syringes lubricated with silicone oil (SiO) are extensively used despite multiple reports showing that such syringes can cause deposition of SiO droplets in the vitreous body and patient discomfort. Thus, there is a need for SiO-free substitutes specifically tailored for IVI. Here, we report on the development and testing of such a syringe. This syringe has no dead volume, and its design allows for high-accuracy dosing. Also, it permits pharmaceutical compounding and storage of bevacizumab, ranibizumab, and aflibercept for up to 30 days without compromising their functional binding or transport properties. Finally, the new syringe demonstrated a favorable safety profile regarding release of SiO compared to SiO lubricated alternatives, including commercially prefilled syringes. Accordingly, the newly developed syringe is an appealing alternative for IVI.

## Introduction

Intravitreal injection (IVI) of anti-vascular endothelial growth factor (anti-VEGF) biologics has revolutionized the treatment and prognosis of several prevalent retinal diseases, such as neovascular age-related macular degeneration (nAMD), diabetic macular edema, and retinal vein occlusion. Despite the extensive use of anti-VEGF therapy in ophthalmology, IVI is typically performed with syringes that are neither developed nor approved for this specific purpose. This brings up particular concerns. First, syringes are commonly coated with silicone oil (SiO), which acts as a lubricant and facilitates smooth plunger movement (1, 2). In the setting of IVI, this may result in deposition of SiO droplets in the vitreous body (3–10), which can cause symptomatic floaters and result in inflammatory responses (11, 12). We have previously reported that among asymptomatic patients, intravitreal SiO was evident in 68% nontheless (10). Second, the material and coating of syringes can affect the stability and concentration of anti-VEGF biologics, in particular upon compounding and storage in prefilled syringes (13–15). Third, anti-VEGF biologics are expensive. As only 0.05 mL is injected intravitreally, the dead space of a syringe can make up a considerable proportion of the total volume withdrawn and thereby increase costs unnecessarily. Finally, different syringes vary with respect to handling and accuracy, which are essential aspects in the face of the high prevalence of IVI (16, 17). Handling differences may also enhance SiO release and particle formation within certain syringes (18, 19).

Here, we report on the development of the Zero Residual™ Silicone Free 0.2 mL Syringe, a new syringe specifically developed for IVI of biologics. We describe the unique features of this syringe and compare its release of SiO to other commercially available alternatives, including commercially prefilled syringes (PFS). In addition, we assess how compounding and storage of anti-VEGF biologics for up to 30 days in the new syringe affects the structural integrity, binding, and transport properties of the biologics.

## Materials and Methods

### Experimental Design

The main objective of this study was to design and develop a new syringe for intravitreal injections followed by a thorough characterization of the properties of the resulting syringe. This characterization consisted of an analysis of SiO release, comparing the new syringe to commercially available syringes, as well as a thorough investigation of the structural integrity and function of the biologics aflibercept, bevacizumab, and ranibizumab after storage for 0, 7, 14, and 30 days in the new syringe, using a methodology comparable to our previous studies (20, 21). During storage, the syringe was either capped or attached to a needle.

The syringe developer (SJJ Solutions) designed the novel syringe based on their current Zero Residual™ small volume 0.3 mL syringe, while taking into account several requirements of ophthalmologists.

### Syringe Preparation in Agitation Study

The syringes were prepared by loading 50 µL of either selected biologics or buffer (60 mg/mL trehalose, 5.8 mg/mL NaH_2_PO_4_, 1.2 mg/mL Na_2_HPO_4_, 0.4 mg/mL polysorbate 20, pH 6.2, 0.22 µm filtered gifted by Vaida Linkuviene, Skaggs School of Pharmacy and Pharmaceutical Sciences, University of Colorado Anschultz) into an Eppendorf tube for individual use and aspirated by the syringe with the appropriate needle attached. The samples from the no-agitation groups were gently handled before the fluid was expelled into a new Eppendorf tube. Syringes in the agitation groups were flicked 15 times with the finger of the same operator in a standardized fashion. The following syringes were included: aflibercept prefilled syringe (PFS) (Eylea, Regeneron Pharmaceuticals), Becton-Dickinson (BD) Ultra-Fine 0.3 mL (Becton-Dickinson and Co.), HSW Norm-Ject 1 mL (Henke Sass Wolf), ranibizumab PFS (Lucentis, Genentech), and Zero Residual™ SFS 0.2 mL (SJJ Solutions). Both HSW Norm-Ject and Zero Residual™ SFS are SiO-free syringes. Aflibercept (Eylea, Regeneron Pharmaceuticals), bevacizumab (Avastin, Roche), and ranibizumab (Lucentis, Genentech) were purchased for the experiments. In order to allow for agitation of the syringe without fluid spillover, a 30-gauge Zero Residual needle (SJJ Solutions) was attached to all syringes, except for the BD Ultra-Fine, which has its own staked-in needle. Each syringe was assessed in triplicate (n=3) for each fluid and condition.

### Fluorescent Labeling

Identification of SiO droplets was performed by fluorescent labeling using the Amnis^®^ Protein Aggregate and Silicone Oil Detection Kit provided by Luminex Corp. Assay buffer and dye were essentially prepared as instructed by the manufacturer. Of note, PMPBF2 was diluted an additional 16-fold for an actual concentration of 10/16X. Samples were fluorescently labeled by adding 3 µL of 10X dye cocktail to 27 µL study sample and then incubated for at least 15 minutes in a dark room prior to data acquisition. Data were acquired within 4 h of preparation as previously described (22). All data were collected using an Amnis FlowSight imaging flow cytometer (IFC) (Luminex), and the data were analyzed using IDEAS^®^ 6.2 (Luminex) image analysis software.

### FlowSight Imaging Flow Cytometry Data Acquisition and Analysis

Imaging data were collected using an IFC equipped with a 20X magnification objective in high-sensitivity mode (flow rate 1.14 mL/min). The 488-nm fluorescence excitation laser and the 785-nm side scatter excitation laser were set to the maximum power at 60 mW and 70 mW, respectively. Brightfield (BF) LED power was automatically set for each sample to achieve a consistent background. BF images were collected in channel 9 (filter 577/35), PMPBF2 images were collected in channel 2 (filter 532/55), ProteoStat images were collected in channel 4 (filter 610/30), and PMPBF2 precipitates were collected in channel 5 (filter 702/86). All identified events were collected (i.e., no user threshold applied). Each sample was measured until a 20,000-particle limit was acquired by the instrument or after a 2-min acquisition time, whichever happened first. Analysis of IFC data was performed in IDEAS^®^ 6.2 image analysis software (MilliporeSigma). SiO droplets were identified according to the analysis template provided by the manufacturer. In brief, dye particles that form through self-aggregation of the PMPBF2 stain, namely PMPBF2 precipitates, were excluded based on red shift. Objects positive for PMPBF2 stain were identified as SiO droplets. The number of SiO droplets measured were converted to particle concentration based on the analyzed volume.

### Concentration Measurements

Samples of aflibercept, bevacizumab, and ranibizumab, prefilled at the Hospital Pharmacy at Oslo University Hospital, were transferred from prefilled syringes to sterile Eppendorf Protein LoBind-tubes (Eppendorf), which were kept on ice and protected from light during the experiments. All samples were diluted 1:10 in sterile phosphate buffered saline (PBS) (Sigma-Aldrich). Protein concentrations were measured using a DeNovix DS-11+ Spectrophotometer (DeNovix). The average values of two measurements per sample were calculated.

### SDS-PAGE Analyses

The protein samples were prepared and analyzed as previously described (20). Briefly, 2 µg protein was diluted in distilled water and Bolt LDS loading buffer (Thermo Fisher Scientific), both with and without DL-dithiothreitol solution (Sigma-Aldrich). Samples with DL-dithiothreitol solution were heated for 5 min at 95°C prior to application to 12% Bolt Bis-Tris Plus gels (Invitrogen) side by side with native samples before running for 22 min at 200 V. Size comparison was performed by using Spectra Multicolor Broad Range Protein Ladder (Fermentas) and the proteins were visualized by Bio-Safe Coomassie G-250 staining (Bio-Rad Laboratories).

### Size Exclusion Chromatography (SEC)

Aliquots from the prefilled syringes were collected and diluted in sterile PBS to 5.6 mg/mL, 3.5 mg/L, and 1.2 mg/mL for aflibercept, bevacizumab, and ranibizumab, respectively. The experiments were performed using an ÄKTA avant 25 (GE Healthcare). As previously reported (20), aflibercept and bevacizumab were run on a Superdex 200 Increase 10/300 GL column (GE Healthcare) and ranibizumab was run on a Superdex 75 Increase 10/300 GL column (GE Healthcare). An auto-sampler (Spark Holland B.V.) was used to inject 77 μL of all samples.

### VEGF Binding Enzyme-Linked Immunosorbent Assay (ELISA)

As described earlier (20), 96-well EIA/RIA 3590 plates (Corning Costar) were coated with 100 μL 0.5 µg/mL human VEGF165 (Sino Biological) and incubated overnight at 4°C. Briefly, the plates were blocked for 2 h with 250 μL 4% skimmed milk powder (S) (Sigma-Aldrich) dissolved in PBS (Sigma-Aldrich) (S/PBS), followed by washing four times with PBS containing 0.05% Tween20 (T) (Sigma-Aldrich). Next, 100 μL of the anti-VEGF biologics were diluted in S/PBS/T and added in a titration series, 1000-0.5 ng for ranibizumab and 2000–0.9 ng for aflibercept and bevacizumab. After incubation at room temperature (RT) for 1 h on a shaker, the plates were washed and 100 μL alkaline phosphatase (ALP)-conjugated goat anti-hFc Ab (Sigma-Aldrich) or ALP-conjugated anti-hKLC diluted to 1 μg/mL in S/PBS/T was added and incubated for 1 h on a shaker for detection. Following washing, the bound proteins were visualized by adding 100 μL ALP substrate (1 mg/mL) dissolved in diethanolamine buffer. The absorbance was measured at 405 nm using a Sunrise spectrophotometer (Tecan Group Ltd.).

### FcRn Binding ELISA

There were 96-well EIA/RIA 3590 plates (Corning Costar) coated with human VEGF165 (Sino Biological) followed by blocking, before titrated amounts of the anti-VEGF biologics were added to the plates as previously described (20). A total of 100 μL of recombinant hFcRn-GST were added at a final concentration of 1 μg/mL diluted in S/PBS/T pH 5.5 (100 mM phosphate buffer, 0.15 M NaCl, 4% skimmed milk, 0.05% Tween 20) or S/PBS/T pH 7.4 and incubated for 1 h at RT on a shaker, as described elsewhere (23). After washing with either pH 5.5 or pH 7.4 PBS/T, horse radish peroxidase-conjugated anti-GST (Rockland Immunochemicals Inc) diluted 1:8000 in either pH 5.5 or pH 7.4 S/PBS/T was added. After incubation for 1 h at RT on a shaker and washing as above, the bound receptor was visualized by adding 100 μL tetramethylbenzidine substrate (Calbiochem). The reaction was stopped by adding 100 μL 1 M HCl. The absorbance was measured at 450 nm using a Sunrise spectrophotometer (Tecan Group Ltd.).

### Human Endothelial Recycling Assay (HERA)

HMEC1 cells stably transfected with human FcRn (HMEC1-hFcRn) (24) were cultivated at 37°C with 5% CO_2_ in MCDB 131 medium (Gibco) containing 25 ug/mL penicillin, 2 mM L-glutamine, 10% heat-inactivated FBS, 1 µg/mL hydrocortisone (Sigma-Aldrich), 10 ng/mL mouse epidermal growth factor (PeproTech), 100 µg/mL G418 (Sigma-Aldrich), and 5 µg/mL blasticidin (*In vivo*Gen). There were 3.75x10^4^ cells seeded in a volume of 250 µL culturing medium in Costar 48-well plates 24 h prior to experiment initiation. Following visual inspection to confirm characteristic morphology and sufficient confluency, the medium was removed from all wells and the cells washed three times in 250 µL RT HBSS (Life Technologies). After washing, cells were starved for 1 h in 250 µL RT HBSS. Next, samples from each syringe were prepared in triplicate, where 800 nM of either aflibercept or bevacizumab diluted in RT HBSS was added to the cells. After 3 h incubation, cells were washed three times in 250 µL ice cold HBSS followed by adding 220 µL RT serum-free culturing medium with MEM non-essential amino acids (ThermoFisher). Finally, recycling samples were harvested after additional 3 h incubation and frozen at -20°C until analysis. Quantification was done by adding the recycling samples to an antigen-binding ELISA as described above. Serial dilutions of 500-0.24 ng/mL of either aflibercept or bevacizumab were included on each ELISA plate, constituting a 12-step standard curve toward which protein levels in the recycling samples were interpolated using a sigmoidal, 4PL regression model. The cells were kept in the incubator both during starvation and the following incubation steps.

### Surface Plasmon Resonance (SPR)

Kinetic measurements of the anti-VEGF biologics toward VEGF were performed by using a Biacore T200 (GE Healthcare) by immobilizing human VEGF165 (Sino Biological) [~300 resonance units (RU)] to CM5 sensor chips using amine-coupling as described by the manufacturer. Briefly, the coupling was performed by injecting 5 µg/mL human VEGF165 dissolved in 10 mM sodium acetate pH 4.5 and using the amine coupling kit (GE Healthcare). HBS-P+ (0.01 M HEPES, 0.15 M NaCl, 0.005% surfactant P20, pH 7.4) was used as both running and dilution buffer. The measurements were performed by injecting 800 nM ranibizumab or 100 nM aflibercept and bevacizumab over the immobilized VEGF165 at a constant flow rate of 30 µL/min (20). To regenerate the CM5 chip between consecutive sample measurements, glycine pH 1.5 (GE Healthcare) was used. The sensorgrams were zero-adjusted and the individual injections normalized using the BIAevaluation software version 4.1 (GE Healthcare).

### Nano-Differential Scanning Fluorimetry (DSF)

Thermal stability of the anti-VEGF biologics was determined by nano-DSF analysis performed on a Prometheus NT.48 (NanoTemper Technologies GmbH). Triplicates of undiluted samples were drawn into capillaries and run on a program where the instrument was set to gradually increase the temperature from 20°C to 95°C. The ratio between 330 nm and 350 nm wavelengths was plotted against temperature as the temperature increased. The melting temperature (Tm) for which half of the proteins were unfolded was determined by deducing the first derivative in the PR.ThermControl software (NanoTemper Technologies GmbH).

### Statistical Analyses of SiO Release

The mean number of SiO droplets and standard deviation for all anti-VEGF biologics and buffer in BD Ultra-Fine, HSW Norm-Ject, and Zero Residual™ SFS are reported, both individually and combined. Data for the aflibercept PFS and ranibizumab PFS, as well as for each individual drug tested, are presented as mean (SD). The comparison of two groups was performed with the unpaired Student *t*-test with Welch’s corrections assuming unequal variance. All displayed p-values are two-tailed. All error bars represent standard deviation (SD) from the sample group mean.

### Statistical Analyses in Stability Studies

Statistical comparison between groups was done by unpaired Student’s *t*-test. D0-n was compared to D0-v to determine whether syringe withdrawal affected the biologics. All other sample groups (D7-n, D7, D14, D30-n, and D30) were compared to D0-n to assess the effect of storage in the syringes. All displayed p-values are two-tailed. ELISA data points for antigen and human FcRn-binding of aflibercept and bevacizumab were excluded if deviating by >30% from group mean in both ELISA set-ups. The same criterion was used for ranibizumab, although calculating from only the antigen-binding ELISA. For statistical comparison of ELISA binding data, n = < 5. For HERA, single wells were excluded if the calculated recycled amount of protein deviated from the intragroup mean by >50%. Regardless, n = < 18 in statistical comparison of the data. All error bars represent standard deviation (SD) calculated from the sample group mean.

## Results

### The Rationale for Syringe Design

**Figure 1:**
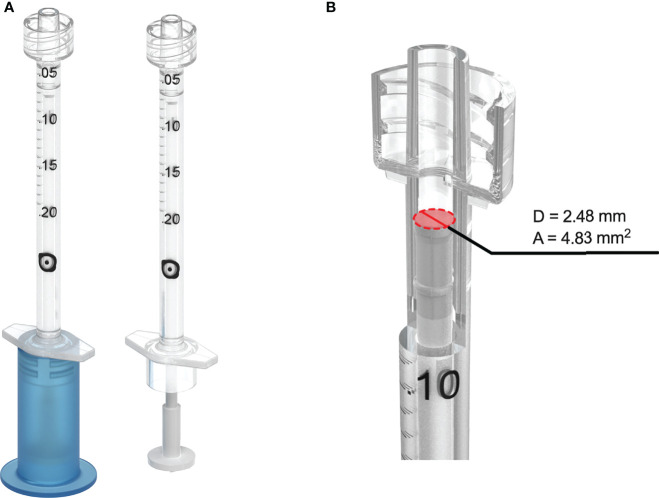
The novel SiO-free syringe. **(A)** Zero Residual™ Silicone Free Syringe (Zero Residual™ SFS) with and without plunger shield attached. **(B)** The barrel of the syringe has a constant inner diameter/area of 2.48 mm/4.83 mm^2^ to allow for high precision injections.

To meet the specific IVI requirements, several innovative elements were incorporated into the syringe design (**Figure 1**). It was designed to be SiO-free and with no dead volume, and the rubber piston was made to mitigate the risk of releasing particles from the barrel caused by friction and movement. It was also designed to maximize sealing ability while maintaining a low break-loose force and constant gliding force. Its total volume was limited to only 0.2 mL, and an injection of 0.05 mL will represent a total displacement of 10.4 mm. A Luer Lock and a dedicated plunger shield were added (**Figure 1A**), which will ensure integrity during transport and storage after being pre-filled with biologics of interest. If local legislation requires the syringe to be capped during storage, the Zero Residual™ Luer Lock cap (SJJ Solutions) can be used to enable the syringe to be prefilled air-free. Additionally, the barrel has a constant inner diameter over the entire length of the syringe and a distal plunger diameter/area of 2.48 mm/4.83 mm^2^ (**Figure 1B**), which will enable dosing accuracy as the volumetric displacement is constant. Along with the high per-volume plunger displacement, this should allow injection with high precision, a minimal pressure on the needle, and a low injection speed compared to the commonly used 1 mL syringes. As such, the designed syringe was given the name Zero Residual™ Silicone Free 0.2 mL Syringe (Zero Residual™ SFS).

### Release of SiO From Syringes

**Figure 2:**
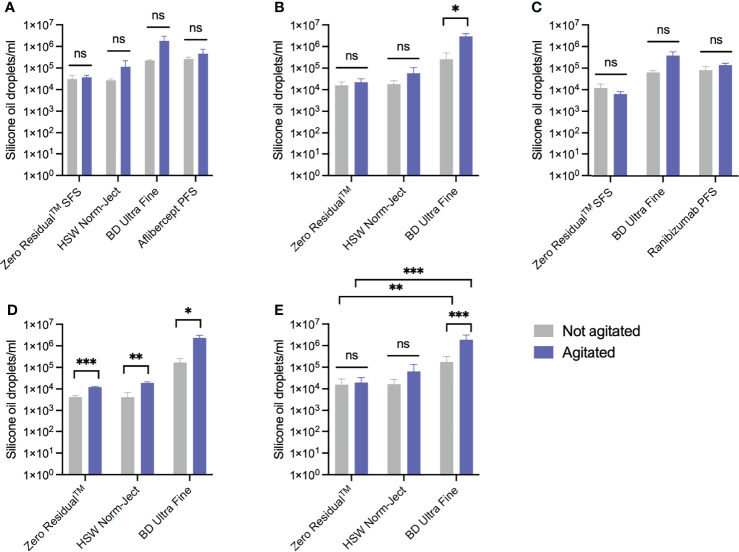
Release of SiO from Zero Residual™ SFS. Release of SiO from the biopharmaceuticals **(A)** aflibercept, **(B)** bevacizumab, and **(C)** ranibizumab from Zero Residual™ SFS and commercially available syringes, where the unagitated syringes are shown in grey and the agitated syringes are shown in purple (n = 3). **(D)** As a control, buffer was run in the same syringes (n = 3). **(E)** The combined results from syringes tested with biologics and buffer (n = 12 for Zero Residual™ SFS and BD Ultra-Fine and n = 9 for HSW Norm-Ject). Unpaired student’s t-test with Welch’s corrections assuming unequal variance was used for statistical analysis and all error bars represent ± SD calculated from the sample group mean. *p < 0.05, **p < 0.005, ***p < 0.0005, ns, not significant.

We compared the release of SiO from Zero Residual™ SFS with that of SiO-containing BD Ultra-Fine (Becton, Dickson and Co.) and SiO-free HSW Norm-Ject (Henke-Sass, Wolf), which are both commonly used syringes for IVI. Additionally, we investigated the SiO release from aflibercept PFS and ranibizumab PFS syringes. As a control, syringes containing buffer were run to investigate if anti-VEGF biologics affected SiO release. Syringes were either agitated by flicking 15 times or handled gently with no agitation. Released SiO droplets were identified by fluorescent labeling and analyzed by imaging flow cytometry (**Table 1** and **Figures 2A–E**).

**Table 1:** Schematic overview of combined SiO release from Zero Residual™ SFS and commercially available syringes.

Syringe	Sample size (n)	Not agitated (particles/ml)	Agitated (particles/mL)	*p*-values
Zero Residual™ Silicone Free Syringe	12	15,804 ± 12,443	19,470 ± 13,448	0.496
HSW Norm-Ject	9	16,439 ± 10,961	63,559 ± 69,414	0.077
BD Ultra-Fine	12	177,816 ± 138,655	1,870,429 ± 1,234,151	0.001
Aflibercept PFS	3	261,243 ± 56,938	464,291 ± 275,350	0.329
Ranibizumab PFS	3	80,758 ± 38,276	138,801 ± 29,207	0.110

Averaged data from all tested biologics and conditions for each syringe. Unpaired student’s t-test with Welch’s corrections assuming unequal variance was used to calculate p-values to compare the various syringes with and without agitation. SD is calculated from the sample group mean.

Interestingly, both aflibercept PFS (**Figure 2A**) and ranibizumab PFS (**Figure 2C**) exhibited notable release of SiO particles even when not agitated, with respective levels of 261,243 ± 56,938 particles/mL and 80,758 ± 38,276 particles/mL (n=3). After agitation, the particle counts increased to 464,291 ± 275,350 particles/mL for aflibercept PFS (**Figure 2A**) and 138,801 ± 29,207 (n=3) for ranibizumab PFS (**Figure 2C**).

The SiO count of Zero Residual™ SFS and HSW Norm-Ject syringes was lower than that of BD UltraFine under all tested conditions. Averaging the SiO count from syringes filled with both buffer and the three tested biologics (bevacizumab 25 mg/mL, ranibizumab 10 mg/mL, and aflibercept 40 mg/mL) revealed that without agitation, the Zero Residual™ SFS released 15,804 ± 12,443 particles/mL (n=12, **Table 1** and **Figure 2E**), a number which increased only slightly to 19,470 ± 13,448 particles/mL after agitation. Similarly, non-agitated HSW Norm-Ject syringes released 16,439 ± 10,961 (n=9) SiO particles, which also showed a small increase after agitation (63,559 ± 69,414 particles/ml). However, for the BD Ultra-Fine syringe, there was a significant increase in the particle count relative to both Zero Residual™ SFS and HSW Norm-Ject (**Table 1** and **Figure 2E**), regardless of agitation state. Notably, BD Ultra-Fine was the only one of the three syringes containing biologics to exhibit significant increased particle release upon agitation, from 177,816 ± 138,655 to 1,870,429 ± 1,234,151 particles/mL (n=12, **Table 1** and **Figure 2E**). Thus, the newly developed syringe demonstrated a favorable safety profile regarding release of SiO compared to SiO lubricated alternatives, including commercially available PFS.

### Compounding in SiO-Free Syringes

To test the novel syringe for compounding of antibody-based anti-VEGF biologics, we adopted a previously established compounding procedure implemented at our hospital pharmacy (20). Briefly, commercially acquired aflibercept, bevacizumab, and ranibizumab were withdrawn into Zero Residual™ SFS under standard aseptic conditions according to ISO 13544 guidelines and EU GMP (25). The syringe was then either attached to a Low Dead Space Needle hub 33 G × 9 mm injection needle (TSK Laboratory) or capped with a Zero Residual™ Luer Lock cap (SJJ Solutions). Each syringe was separately stored at 4°C in dark conditions for 0 days with needle (D0-n), 7 days with both needle (D7-n) and cap (D7), 14 days with cap (D14), and 30 days with needle (D30-n) and cap (D30).

### Protein Concentration and Stability

**Figure 3:**
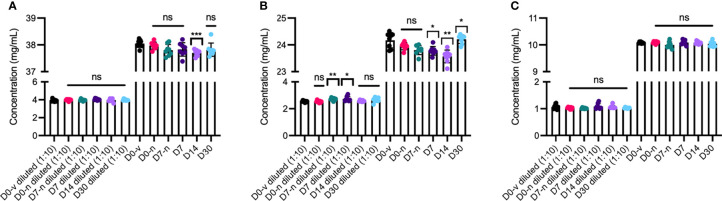
Concentration measurements. Both diluted (1:10) and undiluted samples of **(A)** aflibercept, **(B)** bevacizumab, and **(C)** ranibizumab were measured at Day 0 directly from the vial (D0-v) shown in black, Day 0 from syringe with needle (D0-n) shown in pink, Day 7 with needle (D7-n) shown in teal and cap (D7) in dark purple, Day 14 (D14) and Day 30 (D30) with cap shown in light purple and light blue, respectively. For each sample set n = 8. Unpaired Student’s t-test was used for statistical analysis and all error bars represent ± SD calculated from the sample group mean. *p < 0.05, **p < 0.005, ***p < 0.0005, ns, not significant.

To assess whether syringe compounding and storage affected the sample concentration of the anti-VEGF biologics (n=8), we used spectrophotometry to measure the protein concentrations (**Figure 3**). A minor but significant decrease in concentration was measured between D0-n and D14 for aflibercept (-0.27 mg/mL) (**Figure 3A**), but this was not reproducible for the corresponding diluted sample. Similarly, for bevacizumab, a minor but significant decrease in concentration was measured for both D7 (-0.18 mg/mL) and D14 (-0.38 mg/mL) compared to D0-n, whereas a significant increase was measured for D30 (+0.26 mg/mL) (**Figure 3B**). Importantly, in the diluted samples, both D7-n and D7 had a significantly higher concentration than D0 (+0.18 mg/mL). No differences were observed for ranibizumab (**Figure 3C**). By repeating a smaller sample set (n=4) for aflibercept and bevacizumab with new syringes, comparing D0-n with D7-n, D7, D30-n, and D30 (**Figure S1**), we found no differences for neither diluted nor undiluted samples of aflibercept (**Figure S1A**). In the undiluted samples of bevacizumab, there was a significant increase for D7-n compared to D0-n (+0.32 mg/ml), and significant decreases were measured for both D30-n and D30 (-0.70 mg/ml and -0.70 mg/ml, respectively). However, this was not reproducible in the diluted samples (**Figure S1B**). As such, only minor differences in concentration were measured between samples and the different conditions.

To investigate the protein integrity in the stored syringes, equal amounts of each biologic were added to SDS-PAGE gels under either non-reducing or reducing conditions (n=8) (**Supplementary Figures S2**–**S4**). The migration profiles of the proteins were in line with their expected molecular weights of 96 kDa, 149 kDa, and 50 kDa under non-reducing conditions for aflibercept (**Supplementary Figures S2A–F**), bevacizumab (**Supplementary Figures S3A–F**), and ranibizumab (**Supplementary Figures S4A–F**), respectively. Under reducing conditions, ranibizumab (**Supplementary Figures S4G–L**), migrated as one major band of 25 kDa, corresponding to both the heavy and light chains of the Fab fragment, whereas aflibercept (**Supplementary Figures S2G–L**) and bevacizumab (**Supplementary Figures S3G–L**) migrated as bands with a molecular weight of about 70 kDa and 60 kDa, respectively. Bevacizumab also showed a band corresponding to the light chain of 25 kDa. Importantly, no degradation or aggregation products were detected, and there were no visible differences in integrity between the different time points.

**Figure 4:**
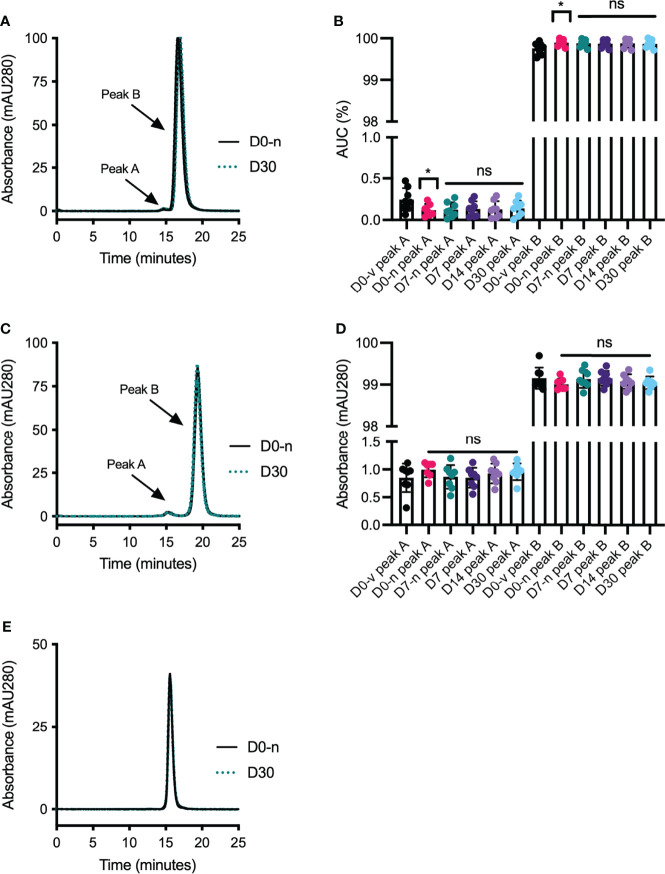
Elution profiles from SEC. Profiles of **(A)** aflibercept, **(C)** bevacizumab, and **(E)** ranibizumab. Profiles for D0-s (black, solid line) and D30 (teal, dotted line) are displayed as start and end-point storage conditions. Area under curve (AUC) was calculated where multiple peaks were observed; for **(B)** aflibercept and **(D)** bevacizumab. D0-v is shown in black, D0-n is shown in pink, D7-n is shown in teal and D7 in dark purple, and D14 and D30 are shown in light purple and light blue, respectively. **(E)** Ranibizumab displayed only one peak. For each sample set n = 8. The data are presented as mean ± SD and unpaired Student’s t-test was used for statistical analysis. *p < 0.05, ns, not significant.

To address if syringe withdrawal and storage may induce formation of non-covalent protein aggregation, we analyzed the anti-VEGF biologics by size exclusion chromatography (SEC). The results showed a major elution peak preceded by a smaller, additional peak for all samples containing aflibercept and bevacizumab (**Figures 4A, C**). In contrast, ranibizumab eluted as a single peak (**Figure 4E**). These findings are identical to previous observations using the same method (20, 21). Statistical comparison of the area under the curve (AUC) demonstrated that syringe withdrawal might have a minor aggregation effect on aflibercept, as indicated by a significant difference between D0-v, directly from the vial, and D0-n in both peak A and B, with a decrease of averaged AUC of peak A (-0.13%) and a slight increase in peak B (0.14%) (**Figure 4B**). However, this effect was not measured for any other time-point, and the difference was considered minor. Comparing AUC values for bevacizumab revealed no protein aggregation (**Figure 4D**). This was also the case for ranibizumab, evident by its single-peak elution (**Figure 4E**).

**Figure 5:**
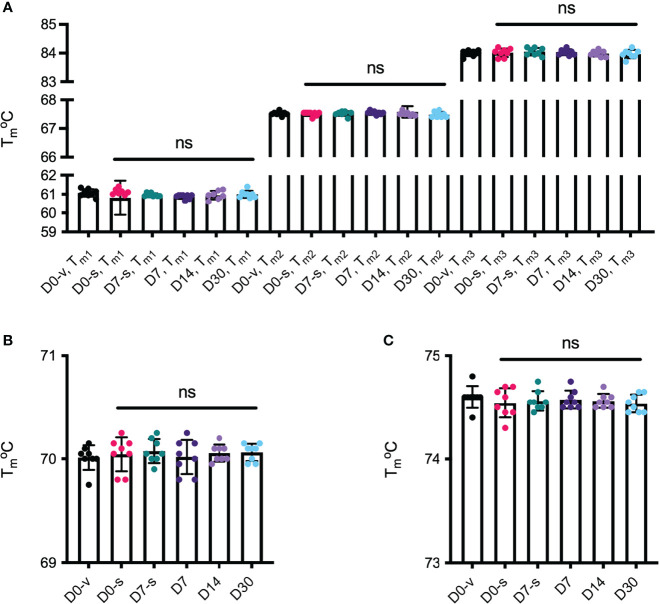
Melting temperatures (Tm°C) of the biologics. Tm°C was measured in nanoDSF for **(A)** aflibercept, **(B)** bevacizumab, and **(C)** ranibizumab. Tm°C for D0-v is shown in black, D0-n is shown in pink, D7-n is shown in teal, D7 is shown in dark purple, and D14 and D30 are shown in light purple and light blue, respectively. The melting process for **(A)** aflibercept occurs in three distinct steps, whereas melting of **(B)** bevacizumab and **(C)** ranibizumab occurred in one event. The data are presented as mean ± SD. For each sample set n = 8, run in triplicates. The unpaired Student’s t-test was used for statistical analysis. ns, not significant.

Last, we determined the thermal stability of the anti-VEGF biologics by nano differential scanning fluorimetry (nanoDSF) (**Figure 5**). The results showed that aflibercept unfolded in three separate events with melting temperature (Tm) values measured at 61°C, 67-68°C, and 84°C (**Figure 5A**), all in accordance with previously published data (20). While bevacizumab showed a Tm of 70°C (**Figure 5B**), ranibizumab gave a slightly higher Tm of 74.5°C (**Figure 5C**) (**Supplementary Tables S1**–**3** for *p*-values). Thus, the thermal stability of the distinct anti-VEGF biologics was unaffected by withdrawal and storage in the Zero Residual™ SFS.

### VEGF Binding Properties

**Figure 6:**
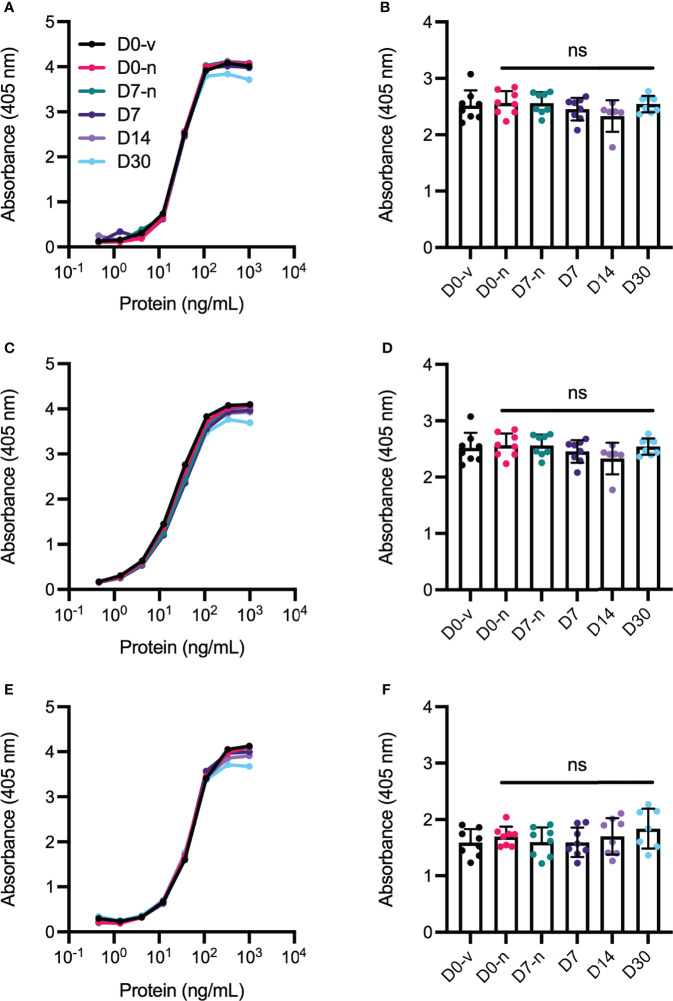
VEGF binding properties determined in ELISA. Titrated amounts of **(A)** aflibercept, **(C)** bevacizumab, and **(E)** ranibizumab added to human VEGF. The binding curves in **(A)**, **(C)**, and **(E)** represent the average of individual binding curves from each sample group (averaged from n = 8 per curve). Single values were retrieved from the exponential phase of the binding curves, at 37.04 ng/mL for statistical comparison, here shown for **(B)** aflibercept, **(D)** bevacizumab, and **(F)** ranibizumab. D0-v is shown in black, D0-n is shown in pink, D7-n is shown in teal, D7 is shown in dark purple, and D14 and D30 are shown in light purple and light blue, respectively. For each sample set n = 8. The data are presented as mean ± SD and unpaired Student’s t-test was used for statistical analysis. ns, not significant.

**Figure 7:**
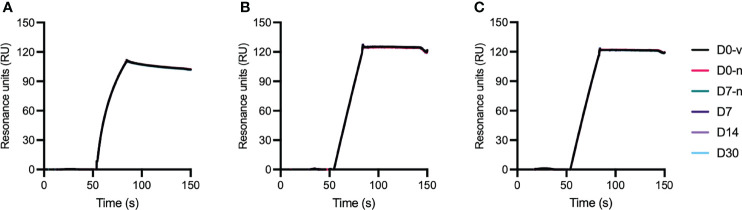
VEGF binding properties in SPR. Sensorgrams showing the binding profiles to immobilized VEGF (~300 RU) for D0-v is shown in black, D0-n is shown in pink, D7-n is shown in teal, D7 is shown in dark purple, and D14 and D30 are shown in light purple and light blue for **(A)** aflibercept, **(B)** bevacizumab, and **(C)** ranibizumab. Each time point is presented as a mean of n = 8 in this overlay. The binding profiles have been normalized to baseline and the blank values subtracted.

The crucial function of anti-VEGF biologics is to effectively bind and neutralize the activity of soluble human VEGF. To assess how the stored biologics bound VEGF, we performed an enzyme linked immunosorbent assay (ELISA) where soluble VEGF was coated in wells followed by addition of titrated amounts of the anti-VEGF biologics. Importantly, no differences were observed in the capacity to bind VEGF (**Figure 6**). This finding was also confirmed by surface plasmon resonance (SPR) experiments where equal amounts of the biologics were injected over immobilized VEGF. Based on overlay of the resulting sensorgrams (**Figure 7**), aflibercept, bevacizumab, and ranibizumab were demonstrated to bind equally well to their cognate antigen, confirming that compounding and storage in the Zero Residual™ SFS did not affect their VEGF binding capacity.

### FcRn Binding Properties

**Figure 8:**
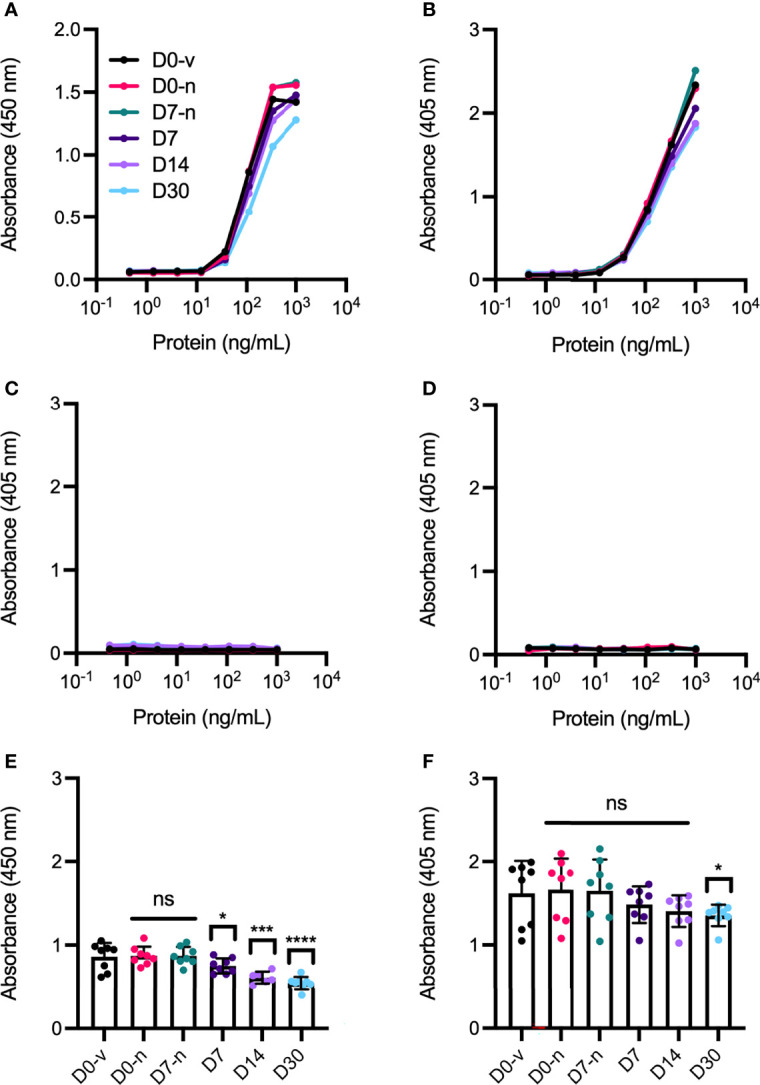
pH dependent human FcRn binding properties of aflibercept and bevacizumab measured in ELISA. Binding of titrated amounts of anti-VEGF biologics to FcRn at pH 5.5 following VEGF capture is displayed in **(A, B)** for aflibercept and bevacizumab, respectively, and pH 7.4 in **(C, D)**. Single values were retrieved from the exponential phase at **(E)** 111.11 ng/mL for aflibercept and **(F)** 333.33 ng/mL for bevacizumab, where D0-v is shown in black, D0-n is shown in pink, D7-n is shown in teal, D7 is shown in dark purple, and D14 and D30 are shown in light purple and light blue, respectively. For each sample set, n = 8. The data are presented as mean ± SD and unpaired Student’s t-test was used for statistical analysis. *p < 0.05, ***p < 0.0005, ****p < 0.00005, ns, not significant.

Antibodies are adaptor molecules that link recognition of their antigen with effector functions mediated by their constant Fc part. One such Fc receptor is the neonatal Fc receptor (FcRn), which regulates transport within and across a range of cells of the body, and is responsible for the long plasma half-life of three weeks at average of full-length IgG antibodies in humans (26). As such, we tested the human FcRn binding properties of the Fc-containing aflibercept and bevacizumab in ELISA, as previously described (20). Since the receptor interacts with the IgG Fc in a strictly pH-dependent manner, with binding at mildly acidic pH (pH 5.5-6.5) and no binding or release at neutral (pH 7.4) (27), the ELISA was performed at both pH conditions (**Figure 8**). The results showed that both aflibercept and bevacizumab bound FcRn in a pH dependent manner, but some reduced binding activity was measured after storage in capped Zero Residual™ SFS. For aflibercept, this reduction was significant for syringes stored with a cap and increased with storage time, displaying a maximal absorbance reduction of 37.5% at D30 (**Figure 8E**). Bevacizumab displayed a less pronounced reduction with a significant difference only detected at D30 (18.7% reduced absorbance) (**Figure 8F**). There was no binding at neutral pH for any of the biologics. We further repeated this particular experiment on a smaller sample set (n=4) for both bevacizumab and aflibercept at acidic pH (**Supplementary Figure S5**). Again, aflibercept and bevacizumab showed reduced receptor binding at D30, both with needle and cap, but this was not significant at the tested concentrations. Thus, compounding and long-term storage of aflibercept and bevacizumab may affect the ability to engage FcRn, an effect that was more pronounced for capped syringes than syringes stored with a needle.

### FcRn-Mediated Cellular Recycling

**Figure 9:**
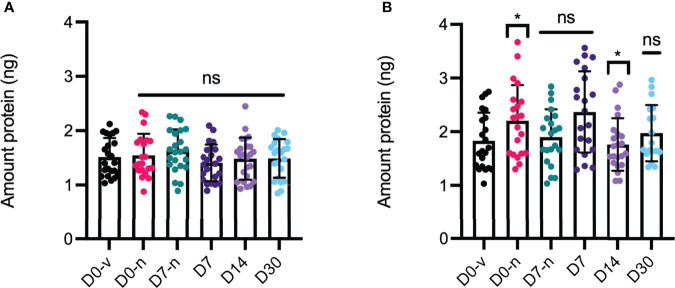
HERA analysis of recycling properties. Amount of protein recycled by microvascular human endothelial cells quantified in subsequent VEGF-capture ELISA for **(A)** aflibercept and **(B)** bevacizumab. D0-v is shown in black, D0-n is shown in pink, D7-n is shown in teal, D7 is shown in dark purple, and D14 and D30 are shown with cap in light purple and light blue. For each sample set n = 8. *p < 0.05, ns, not significant.

Plasma half-life extension of IgG Fc-containing molecules is mediated by FcRn and its pH-dependent cellular recycling process (28). To evaluate the ability of the anti-VEGF biologics to be rescued from intracellular degradation in an FcRn-dependent manner, we took advantage of a recently developed human endothelial recycling assay (HERA) (29). Briefly, anti-VEGF samples from the syringes were added to HMEC1 cells stably expressing human FcRn (24), incubated for 3 h to allow for cellular uptake followed by replacement of the samples with serum-free culturing medium supplemented with non-essential amino acids. Then, the cells were incubated for three additional hours to allow for FcRn-dependent recycling of the Fc-containing biologics from the cells, measured as release into the medium. The amounts rescued from intracellular degradation were quantified by adding the harvested media to the anti-VEGF-specific ELISA as described above (**Figure 9**). As the biologics were captured on coated VEGF, this set-up measures recycling of fully functional aflibercept and bevacizumab. The results revealed that none of the storage conditions affected the ability of aflibercept to be rescued from intracellular degradation (**Figure 9A**). In contrast, a slight but significant increase in recycling was measured for samples taken on D0-n compared to D0-v (+0.38 ng) for bevacizumab, whereas a slight decrease (-0.45 ng) in the recycling capacity was observed for storage up to D14 (**Figure 9B**). Notably, this observation was significant for D14, but the total recycled amounts were nearly identical to those observed for D0-v. As such, the differences in recycled amounts of the anti-VEGF biologics were minor. Thus, the Fc-containing proteins bound human FcRn in a pH-dependent manner and were efficiently recycled in a cellular system.

## Discussion

Intravitreal anti-VEGF therapy is extensively used in contemporary ophthalmology, but typically performed with syringes that are not approved for ophthalmic use. We specifically developed a novel syringe, the Zero Residual™ SFS, to meet the particular demands of IVI, and studied its properties. Zero Residual™ SFS is SiO-free, and correspondingly we found a significantly lower SiO particle release from the novel syringe than common SiO-containing syringes, including commercially prefilled syringes with ranibizumab or aflibercept.

Most commercially available syringes routinely used for IVI have been developed for use in other clinical settings and have not been methodically tested for ophthalmic use. Still, we have previously reported a method for pharmaceutical compounding of anti-VEGF antibody-based biologics for IVI, with the use of a commercially available SiO-free syringe and a low dead-space needle (20). We demonstrated that anti-VEGF biologics could be stored in this syringe for one week without compromising the functional activity of the proteins (20). Nonetheless, the syringe used was not designed for IVI as it lacks a Luer Lock connection and uses a polyethylene based plunger-stopper, causing plastic-on-plastic friction. Consequently, tactile feedback control during the IVI procedure is diminished, and the risk of releasing particles other than SiO possibly increased (1, 18). We report on the development and testing of the Zero Residual™ SFS, a syringe specifically designed for compounding and storage of biologics for IVI. The novel syringe allows for compounding without drug wastage, as well as storage and safe transportation of prefilled syringes. The syringe design also allows for high dosing accuracy and additionally contains a Luer Lock for a safe injection technique. Our study confirms the advantage of an SiO-free design with a significant difference in SiO particle release between SiO-free and SiO-containing BD Ultra-Fine insulin syringes. Furthermore, the study reinforces the prior finding that agitation by flicking to remove air bubbles or other means of mechanical shock, for example during transportation, enhances release of SiO (5, 18, 19, 30–32). In January 2021, the manufacturer of the BD Ultra-Fine remarkably issued a security statement emphasizing that their SiO-containing syringes were not validated for IVI (33). Intriguingly, we found that both aflibercept PFS and ranibizumab PFS also released a significant number of SiO droplets, even in an unagitated state. One can argue that company prefilled syringes are practical. Still, our study confirms that even PFS syringes harbor a risk of releasing SiO during IVI (34). There are also recent reports of transient central retinal artery occlusions due to a possible variability in expressed fluid volumes and incorrect handling of aflibercept PFS syringes (16, 35).

When we measured protein concentration in the Zero Residual™ SFS after compounding and storage, only small differences were detected for aflibercept and bevacizumab. These differences could be due to aggregation. However, no aggregation was detected on the SDS-PAGE gels or during SEC analysis. Binding to the cognate antigen, VEGF, was also shown to be unaffected by compounding and storage. However, for both aflibercept and bevacizumab, we did detect a significant reduction in human FcRn binding at acidic pH for the Fc-containing proteins when derived from the capped syringes. This may be a result of protein oxidation, as oxidation of amino acid residues in the human IgG1 Fc has been shown to reduce human FcRn binding (36). The first set of experiments was conclusive for ranibizumab, but the concentration measurements and FcRn-binding for aflibercept and bevacizumab raised questions. As such, experiments for the two were repeated; no differences in concentration were observed in diluted and undiluted aflibercept samples, but there were significant differences for bevacizumab in the undiluted samples. We also repeated the ELISA for binding to human FcRn, in which the previous significant differences were eliminated at a given concentration in the exponential phase. Yet, the data showed a clear trend toward reduced binding with increasing storage time. However, and importantly, this did not impair their ability to be rescued from intracellular degradation by FcRn in a cellular assay (HERA). As such, the reduced binding responses measured in ELISA, when the biologics were captured on coated soluble VEGF, did not translate into differences in cellular recycling. In summary, we demonstrated that the compounding and storage conditions did not cause aggregation or affect antigen binding for the three anti-VEGF biologics tested, neither in capped syringes nor with needle attached. Additionally, we found that the compounding and storage procedures did not impair the cellular recycling of Fc-containing anti-VEGF biologics.

This study has limitations that should be noted. First, needles used for IVI are generally siliconized to promote scleral penetration, and we used a standard SiO-coated needle in all experiments. The needle is the likely source of the SiO traces we measured in the SiO-free syringes. Second, the Zero Residual™ SFS is not yet commercially available, which prevented us from performing a cost analysis. However, given the minimal residual volume after injection, the syringe should enable effective and efficient use of costly anti-VEGF drugs.

In conclusion, the Zero Residual™ SFS, an SiO-free syringe tailored for IVI administration of anti-VEGF biologics, can be safely used for compounding and storage of aflibercept, bevacizumab, and ranibizumab for up to 30 days without compromising their biological properties.

## Data Availability Statement

The raw data supporting the conclusions of this article may be made available by the authors upon reasonable request.

## Author Contributions

TG: conceptualization, formal analysis, investigation, visualization, writing – review and editing, HL: conceptualization, formal analysis, investigation, visualization, writing – original draft, writing – review and editing, GM: conceptualization, formal analysis, investigation, resources, writing – review and editing, SM: investigation, CP: formal analysis, investigation, MS: conceptualization, writing – review and editing, ØJ: conceptualization, writing – review and editing, MM: conceptualization, supervision, resources, writing – original draft, writing – review and editing, JA: conceptualization, supervision, resources, writing – original draft, writing – review and editing. All authors contributed to the article and approved the submitted version.

## Funding

This work was supported by South-Eastern Norway Regional Health Authority grant no 2019047 (HL), Dr. Jon S Larsens Foundation (HL), The Norwegian Association of Blind and Partially Sighted (HL), Internal funding from the Division of Head, Neck and Reconstructive Surgery, Oslo University Hospital (TG).

## Conflict of Interest

GM, ØJ, MS, JA, and MM are part of collaborative agreements related to development of the syringes by SJJ Solutions. MM has been a member of advisory boards at Bayer, Roche, Novartis, and Allergan and has received lecture fees from Bayer and Roche. ØJ has been a member of advisory boards at Bayer, Roche, and Allergan and has received lecture fees from Bayer, Roche, and Allergan. Author CP was employed by Luminex Corporation.

The authors declare that this study received funding from SJJ Solutions. The funder had the following involvement: SJJ Solutions designed the Zero Residual™ Silicone Free Syringe in collaboration with the researchers, and was responsible for producing the syringe.

## Publisher’s Note

All claims expressed in this article are solely those of the authors and do not necessarily represent those of their affiliated organizations, or those of the publisher, the editors and the reviewers. Any product that may be evaluated in this article, or claim that may be made by its manufacturer, is not guaranteed or endorsed by the publisher.

## Abbreviations

IVI, intravitreal injections; VEGF, vascular endothelial growth factor; SiO, silicone oil; nAMD, neovascular age-related macular degeneration; PFS, prefilled syringe; Zero Residual™ SFS, Zero Residual™ Silicone Free 0.2 mL Syringe; SEC, size exclusion chromatography; AUC, area under the curve; Tm, melting temperature; nanoDSF, nano differential scanning fluorometry; ELISA, enzyme-linked immunosorbent assay; SPR, surface plasmon resonance; FcRn, neonatal Fc-receptor; HERA, human endothelial cell-based recycling assay.
