# Supplementary material for: A Silicone Oil-Free Syringe Tailored for Intravitreal Injection of Biologics
Source: Front Ophthalmol (Lausanne). 2022 May 4;2:882013. doi: 10.3389/fopht.2022.882013 (PMC11182194; doi:10.3389/fopht.2022.882013)
Supplement: Supplementary file 1 [file DataSheet_1.docx]

Supplementary Material

**This PDF file includes**

Supplementary Text

Figures S1 to S5

Table S1-S3

Supplementary Text

Figure S1 are results from a repeated smaller sample set for aflibercept and bevacizumab, showing concentration measurements. Figures S2-S4 are showing the complete set of SDS-PAGE gels run for aflibercept, bevacizumab and ranibizumab, respectively, under both non-reducing (A-F) and reducing conditions (G-L), in line with previously published analyses of these biologics^20^. Table S1-S3 are overviews of T_m_°C from nanoDSF for the three separate biologics. Figure S5 are results from the repeated smaller sample set for aflibercept and bevacizumab, showing FcRn binding properties.


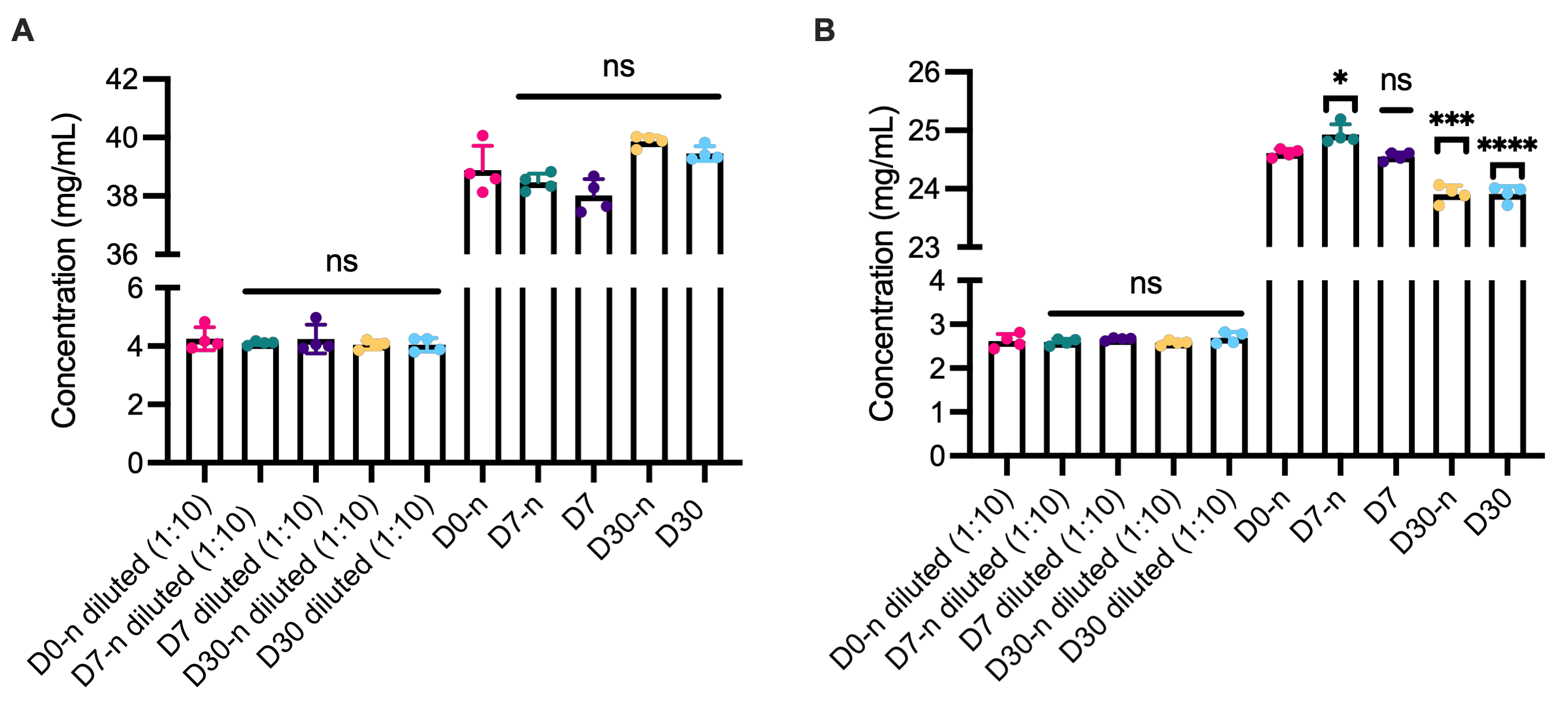


**Figure S1.** Concentration measurements of both diluted (1:10) and undiluted samples for **(A)** aflibercept and **(B)** bevacizumab where D0-n is shown in pink, D7-n shown in teal and D7 in dark purple, D30-n in yellow and D30 with cap shown in light blue. For each sample set n=4. The data are presented as mean ± SD and unpaired Student’s t-test was used for statistical analysis. *p<0.05, ***p<0.0005, ****p<0.00005, ns: not significant.


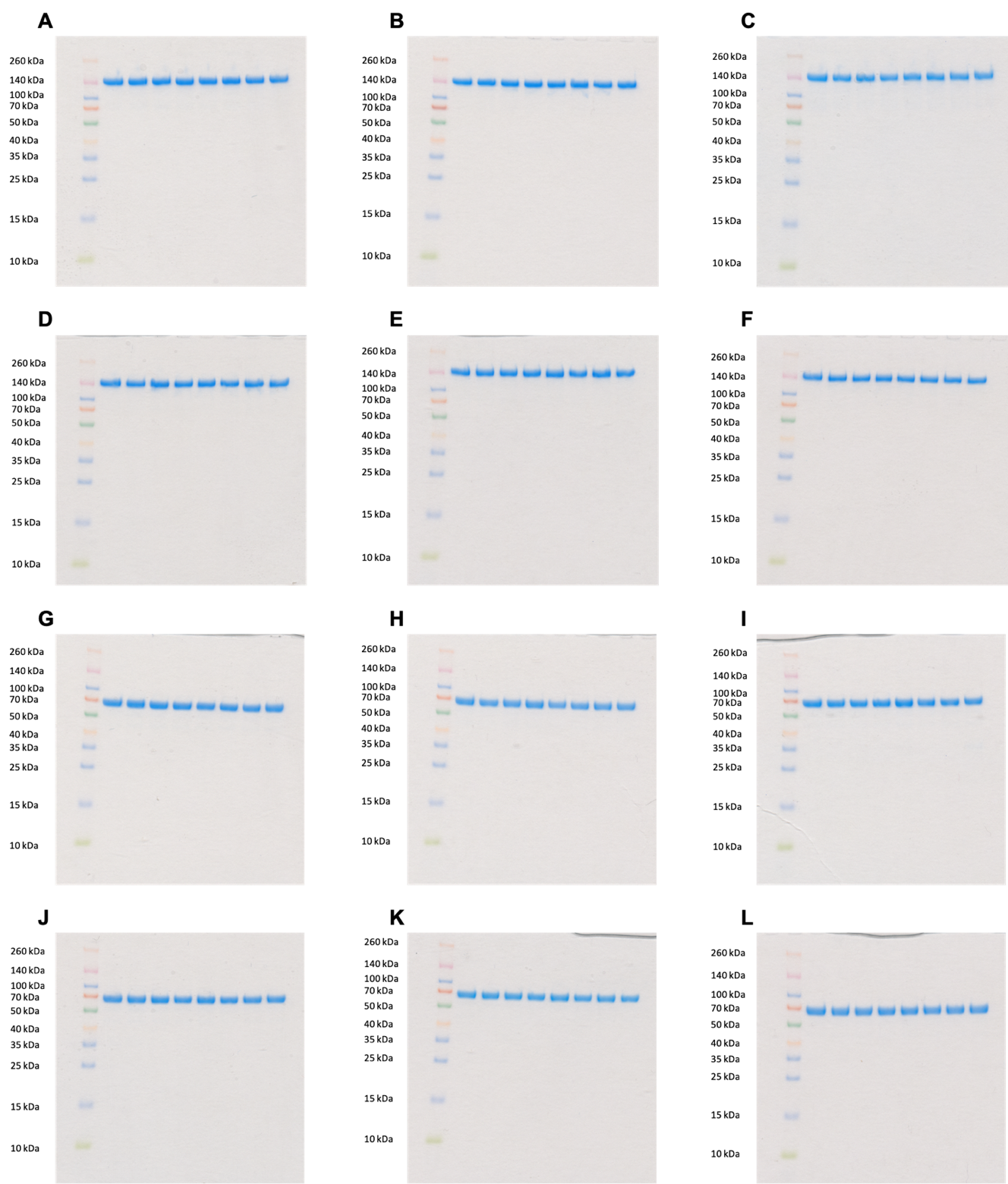


**Figure S2.** SDS-PAGE analysis of aflibercept for **(A)** D0-v, **(B)** D0-n, **(C)** D7-n, **(D)** D7, **(E)** D14 and **(F)** D30 under non-reducing conditions and **(G)** D0-v, **(H)** D0-n, **(I)** D7-n, **(J)** D7, **(K)** D14 and **(L)** D30 under reducing conditions. For each sample set n=8. The gels have not been cropped and only the border of the scanned images edited out.


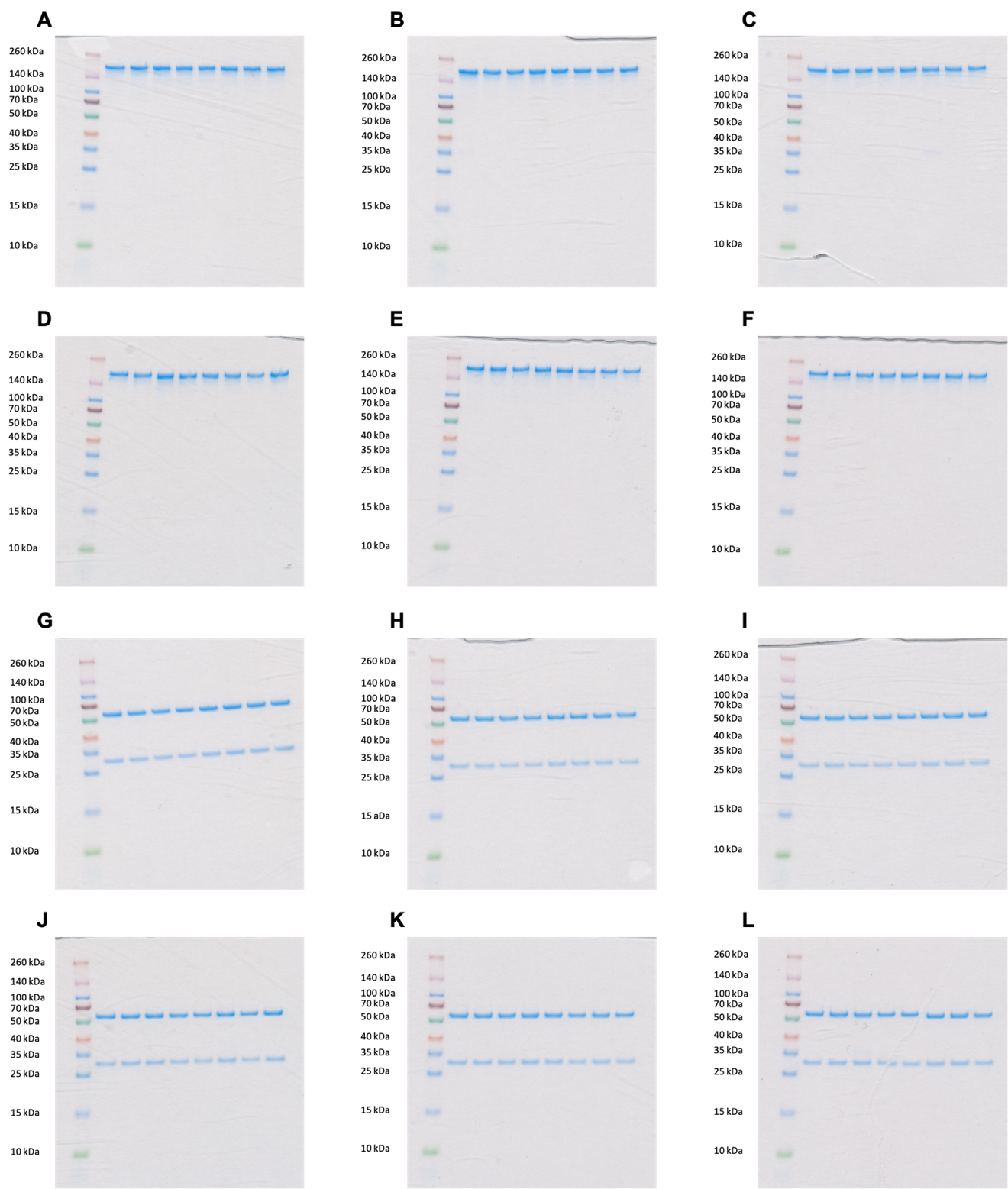


**Figure S3.** SDS-PAGE analysis of bevacizumab for **(A)** D0-v, **(B)** D0-n, **(C)** D7-n, **(D)** D7, **(E)** D14 and **(F)** D30 under non-reducing conditions and **(G)** D0-v, **(H)** D0-n, **(I)** D7-n, **(J)** D7, **(K)** D14 and **(L)** D30 under reducing conditions. For each sample set n=8. The gels have not been cropped and only the border of the scanned images edited out.


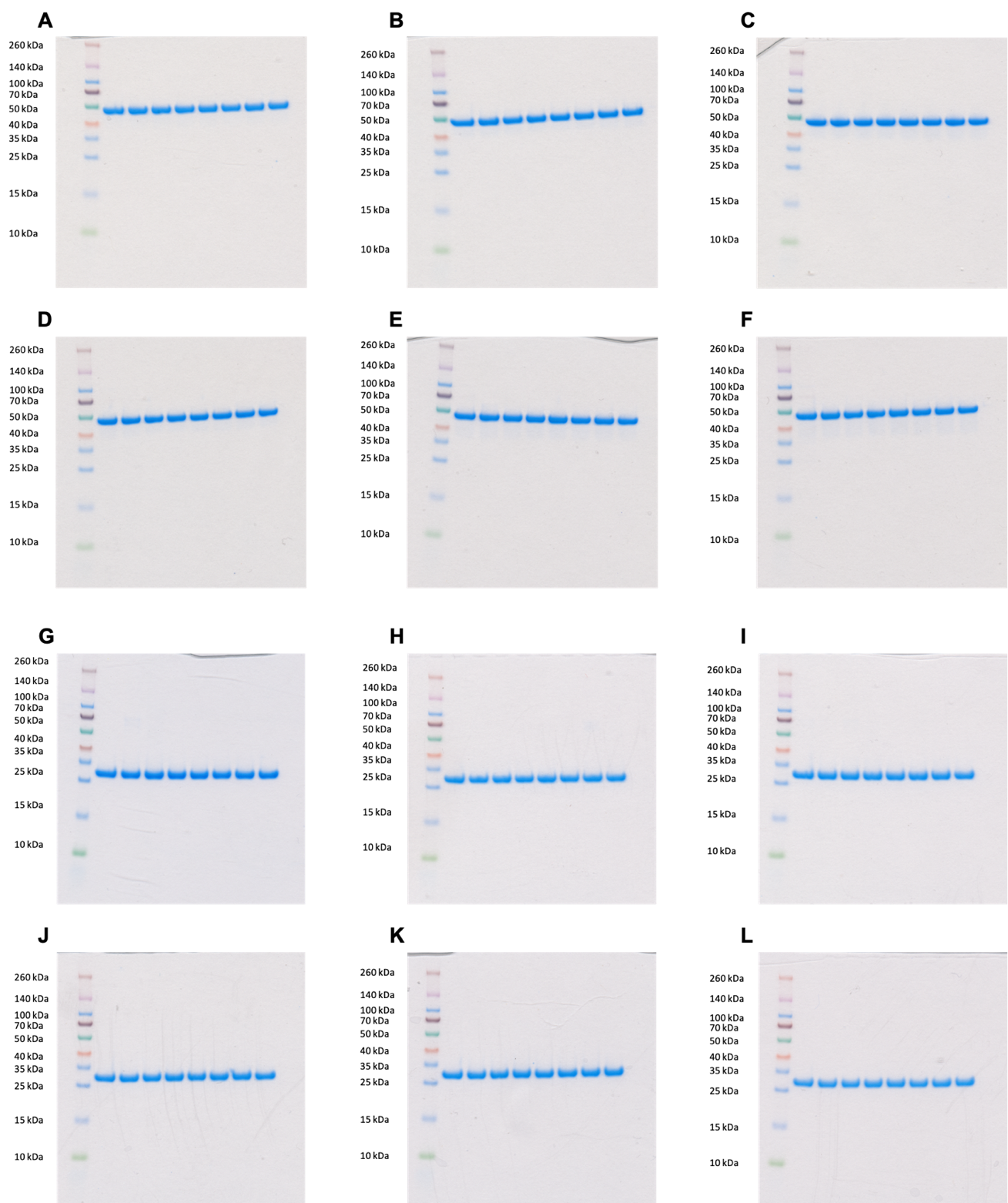


**Figure S4.** SDS-PAGE analysis of ranibizumab for **(A)** D0-v, **(B)** D0-n, **(C)** D7-n, **(D)** D7, **(E)** D14 and **(F)** D30 under non-reducing conditions and **(G)** D0-v, **(H)** D0-n, **(I)** D7-n, **(J)** D7, **(K)** D14 and **(L)** D30 under reducing conditions. For each sample set n=8. The gels have not been cropped and only the border of the scanned images edited out.


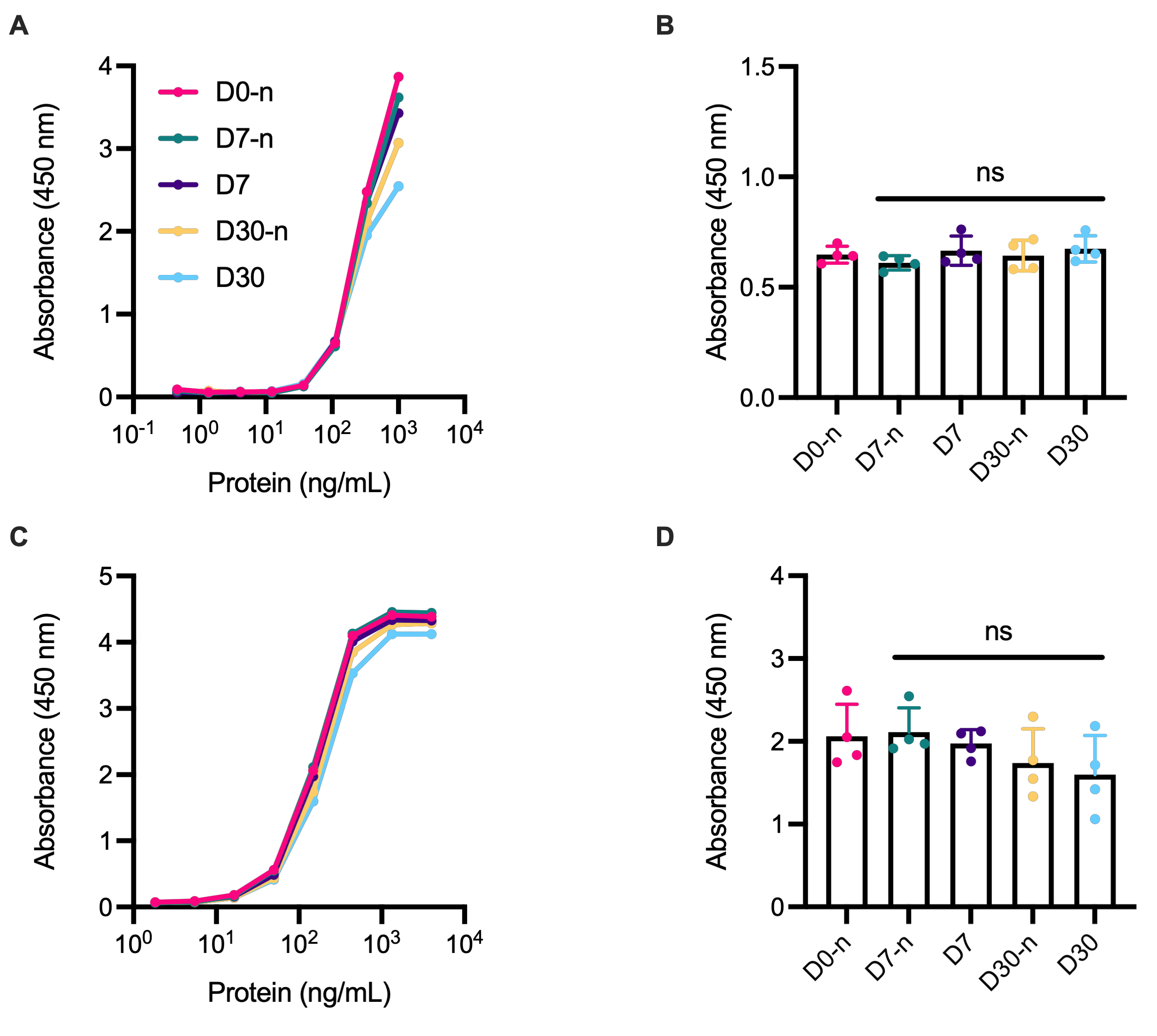


**Figure S5.** pH dependent human FcRn binding properties of aflibercept and bevacizumab measured in ELISA. Binding at pH 5.5 is displayed in **(A)** and **(C)** for aflibercept and bevacizumab, respectively. Single values were retrieved from the exponential phase at **(B)** 111.11 ng/mL for aflibercept and **(D)** 148.15 ng/mL for bevacizumab where D0-n is shown in pink, D7-n shown in teal and D7 in dark purple, D30-n in yellow and D30 with cap shown in light blue. For each sample set n=4. The data are presented as mean ± SD and unpaired Student’s t-test was used for statistical analysis. *p<0.05, ***p<0.0005, ****p<0.00005, ns: not significant.

| **Aflibercept** | T_m_1°C | T_m_2°C | T_m_3°C |
| --- | --- | --- | --- |
| D0 vial – D0 syringe | 0.4363 | 0.7356 | 0.8477 |
| D0 syringe – D7 syringe | 0.6481 | 0.7513 | 0.6001 |
| D0 syringe – D7 | 0.8344 | 0.2851 | 0.7709 |
| D0 syringe – D14 | 0.7035 | 0.3774 | 0.7039 |
| D0 syringe – D30 | 0.5757 | 0.4893 | 0.5618 |

Table S1. An overview of the p-values from the unpaired Student’s *t*-test used for statistical analysis. For each sample set n = 8, measured in triplicates.

| **Bevacizumab** | T_m_°C |
| --- | --- |
| D0 vial – D0 syringe | 0.6685 |
| D0 syringe – D7 syringe | 0.6665 |
| D0 syringe – D7 | 0.7650 |
| D0 syringe – D14 | 0.8496 |
| D0 syringe – D30 | 0.7769 |

Table S2. An overview of the p-values from the unpaired Student’s *t*-test used for statistical analysis. For each sample set n = 8, measured in triplicates.

| **Ranibizumab** | T_m_°C |
| --- | --- |
| D0 vial – D0 syringe | 0.3869 |
| D0 syringe – D7 syringe | 0.7617 |
| D0 syringe – D7 | 0.6067 |
| D0 syringe – D14 | 0.7429 |
| D0 syringe – D30 | 0.9174 |

Table S3. An overview of the p-values from the unpaired Student’s *t*-test used for statistical analysis. For each sample set n = 8, measured in triplicates.
